# ABO groups can play a role in susceptibility and severity of COVID-19

**DOI:** 10.1186/s43168-020-00051-w

**Published:** 2021-02-03

**Authors:** S. Samra, M. Habeb, R. Nafae

**Affiliations:** 1 Zagazig University, Zagazig, Egypt; 2 KFH-Almadina Almonoura, Riyadh, Kingdom of Saudi Arabia

**Keywords:** COVID-19, ABO blood groups, Severity, Mortality

## Abstract

**Background:**

A few people infected by the coronavirus become seriously ill, while others show little to no signs of the symptoms, or are asymptomatic. Recent researches are pointing to the fact that the ABO blood group might play an important role in a person’s susceptibility and severity of COVID-19 infection. Aim of the study: try to understand the relationship between ABO groups and COVID-19 (susceptibility and severity).

**Results:**

A total of (507) patients were included in this study. The study population was divided based on the ABO blood group into types A+, A−, B+, AB, O+, and O−. Blood group A was associated with high susceptibility of infection: group A, 381 (75.1%); and less common in group O, 97 (19.2%), group B, 18 (3.5%), and group AB, 11 (2.2%). The severity of COVID-19 infection was common in non-blood group O where (20 (7.1%), 4 (26.7%), 2 (11%), and 1 (9%) in type A+, A−, B+, and AB, respectively), while in type O 3.1%. And mechanically ventilated patients were 22 (5.9%), 2 (13.4%), 2 (11.1%), and 1 (1%). Mortality was high in blood groups A and B, 16 (4.37%) and 1 (5.5%), respectively, while in blood group O, it was 1%.

**Conclusion:**

The incidence, severity, and mortality of COVID-19 were common in non-blood group O. While blood group O was protected against COVID-19.

## Background

The rapid global spread of the novel coronavirus SARS-CoV-2 has strained existing healthcare and testing resources, and it is causing COVID-19 to hit some people harder than others, with some people experiencing only mild symptoms and others being hospitalized and requiring ventilation. Making the identification and prioritization of individuals most at risk a critical challenge.

The pathogenesis of severe COVID-19 and the associated respiratory failure are still unclear, but the higher mortality is consistently tied to older age and being male. Further, people with underlying health conditions are more likely to develop severe COVID-19, including hypertension, diabetes, being obese, and cardiovascular disease [1]. Biological factors that determine susceptibility to SARS-CoV-2 and severity of COVID-19 are yet to be fully understood. Many studies have implicated that the ABO blood type is a potential risk for various diseases such as cancer, myocardial infarction, acute kidney injury, and venous thromboembolism [2, 3]. The ABO blood grouping may influence the susceptibility of COVID-19 and the severity of the disease [4]. The aim of the study is to study if there is a relationship between the ABO groups and COVID-19 (susceptibility and severity).

## Methods

The study was conducted on 507 COVID-19-confirmed patients admitted in the King Fahd Hospital Almadina Almonoura, KSA, and approved with ethics approval and consent to participate of King Fahd Hospital-Medina, KSA. The written consent from the patients was taken; 401 patients (79%) were admitted in isolation wards while 106 patients (21%) in the ICU, with a mean age ± SD of 54.6 ± 9 with a male to female ratio of 303 (59.7%) and 204 (40.3%), respectively, from the 15 April 2020 to 30 July 2020.

All patients underwent the following:

1. Through medical history: especially smoking history and any comorbid condition such as hypertension and diabetes mellitus.
2. The diagnosis of COVID-19 was confirmed by a positive real-time reverse transcriptase polymerase chain reaction test of SARS-CoV-2 on nasal and pharyngeal swab specimens from patients.
3. ABO grouping is done through blood grouping reagent anti-monoclonal diagnostic test kit. Mediclone Biotech Private Limited, India.
4. Determine the severity of COVID-19 infection. According to ACEP2020 [5] divided patient condition into the following:
  - Mild low risk
  - Mild at risk
  - Moderate
  - Severe
  - Critical
5. Follow up the patients to know the prognosis of the disease cured or died (from 14 to 39 days).

Inclusion criteria:

- Any COVID-19 patient agrees to participate in the study

Exclusion criteria:

- The age of COVID-19 patient is less than 18 years.

### Statistical analysis

An analysis, using SPSS version 12, was performed with respect to the main study aim. Descriptive characteristics for participants are expressed as means and standard deviation (SD) for continuous variables, and number and percentage for categorical variables. We used the independent sample test to show the significant difference between the continuous variable and the chi-square test for the categorical variables. The level of significance was accepted at *p* ≤ 0.05.

## Results

In Table 1, COVID-19 was common in males than in females (303 (59.7%) and 204 (40.2%), respectively). Most of the COVID-19 patients were mild to moderate smoker while heavy and nonsmoker was less common (58.4%, 16.5%, 25%). HTN, DM, IHD, and CVA were the most common comorbid among COVID-19 patients (48.1%, 47.5%, 27.8%, 37.8%, respectively) while chronic chest diseases (bronchial asthma, bronchiectasis) were less common (5.1%, 2.5%, respectively). Most of the COVID-19 patients were mild to moderate cases (449 patients (88.6%)), while critical patients were 36 (7.1%), mechanically ventilated patients were 28 patients (5.5%), and about 4% of the patients died, mostly patients who were in blood group A 16/18 patients (88.9%).

**Table 1:** Some epidemiological and clinical parameters among the ABO group

	A+ (366)	A− (15)	B+ (18)	AB+ (11)	O+ (94)	
**Age**	54.5 ± 11	51.9 ± 4	52.1 ± 6	52 ± 9	54.7 ± 8	53.2 ± 7
**Sex**						
**Male**	212	9	11	6	63	2
**Female**	154	6	7	5	31	1
**BMI**	25 ± 4	24.5 ± 5	24 ± 3	23 ± 7	24.1 ± 5	24.4 ± 6
**Smoking history**						
**Non**	87 (23.7%)	4 (26.7%)	7 (38.9%)	4 (36.3%)	24 (25.5%)	1 (33.4%)
**Mild**	128 (34.9%)	5 (33.3%)	4 (22.3%)	3 (27.2%)	33 (35.1%)	1 (33.4%)
**Moderate**	90 (24.5%)	3 (20%)	4 (22.3%)	3 (27.2%)	21 (22.3%)	1 (33.3%)
**Heavy**	61 (16.7%)	3 (20%)	3 (16.7%)	1 (9.3%)	16 (17.1%)	–
**Comorbidity**						
**No comorbidity**	57 (15.5%)	3 (20%)	3 (16.7%)	4 (36.3%)	41 (43.6%)	1 (33.3%)
**HTN**	202 (55.2%)	8 (53.4%)	6 (33.4%)	8 (72.7%)	20 (21.2%)	–
**DM**	198 (54%)	6 (40%)	5 (27.8%)	8 (72.7%)	22 (32.4%)	2 (66.7%)
**IHD**	119 (32.5%)	3 (20%)	2 (11%)	2 (18.1%)	15 (15.9%)	–
**CVA**	177 (48.3%)	2 (13.4%)	2 (11%)	4 (36.2%)	7 (7.4%)	–
**Chronic chest disease**	25 (6.8%)	1 (6.7%)	–	1 (9%)	12 (12.7%)	–
**Bronchial asthma**	17 (68%)	1 (100%)	–	–	8 (66.7)	–
**Bronchiectasis**	8 (32%)	–	–	1 (100%)	4 (33.3%)	–
**Severity of COVID-19**						
**Mild low risk**	280 (76.5%)	6 (40%)	9 (50%)	3 (27.2%)	51 (54.2%)	2 (66.7%)
**Mild at risk**	18 (4.9%)	1 (6.7%)	2 (11.1%)	3 (27.2%)	23 (24.4%)	–
**Moderate**	30 (8.1%)	1 (6.7%)	3 (16.7%)	3 (27.2%)	10 (10.6%)	1 (33.3%)
**Severe**	12 (3.2%)	3 (20%)	1 (5.5%)	1 (9%)	5 (5.3%)	–
**Critical**	26 (7.1%)	4 (26.7%)	2 (11.1%)	1 (9%)	3 (3.1%)	–
**MV**	22 (5.9%)	2 (13.4%)	2 (11.1%)	1 (9%)	1 (1%)	–
**Outcome**						
**Died**	16 (4.37%)	–	1 (5.5%)	–	1 (1%)	–

*BMI* body mass index, *HTN* hypertension, *DM* diabetes mellitus, *IHD* ischemic heart diseases, *CVA* cerebro-vascular stroke, *MV* mechanical ventilation

In Table 2, there were no significant differences between blood group A and blood group O regarding the age of the patients, male/female, and BMI of the patients [(54.3 ± 9, 55.3 ± 7), (58/42%, 67/33%), (24.7 ± 6, 24.9 ± 8), respectively]. There was no significant difference between the two groups regarding smoking index (mild, moderate, and heavy: 34.9/35%, 24.4/22.6%, 16.8/16.5%, respectively) while there was a significant increase in comorbid condition HTN, DM, IHD, CVA, and chronic chest diseases such as bronchial asthma and bronchiectasis [(55.1%, 20.6%), (53.5%, 24.7%), (32%, 15.5%) and (46.9%, 7.2%) (26/12 patients) and (8/4 patients), respectively]. There was a significant increase in the severe and critical patents in blood group A than in blood group O (15/5, 30/3, respectively). Mechanically ventilated and dead patients have a significant increase in blood group A than in blood group O (24/2, 16/1, respectively).

**Table 2:** Comparison between blood group A and blood group O regarding epidemiological characters of COVId19 patients

	Blood group (A)(381)	Blood group (O)(97)	Test of sig T	*P* value
Age (years) Mean ± SD	54.3± 9	55.3± 7	0.89	0.154
BMI (Mean ± SD)	24.7± 6	24.9± 8	0.9	0.12
	NO /%	No /%	χ2	P value
Male	221 (58%)	65(67%)	1.4	0.21
Female	160 (42%)	• *32(33%)*	0.19	0.14
Smoking History:				
Non	91(23.8%)	25(25.7%)	1.24	2.14
Mild	• *133(34.9%)*	34(35%)	2.01	1.25
Moderate	93(24.4%)	22(22.6%)	0.35	1.58
Heavy	64(16.8%)	16(16.5%)	1.02	0.687
Comorbidity				
No comorbid	60(15.7%)	42(43.2%)	0.357	0.241
HTN	210(55.1%)	20(20.6%)	4.5	0.001**
DM	204(53.5%)	24(24.7%)	9.7	0.00**
IHD	122(32%)	15(15.5%)	5.3	0.001**
CVA	179(46.98%)	7(7.2%)	11.4	0.001**
Chronic Chest dis	26(6.8%)	12(12.3%)	8.7	0.00**
Br. asthma.	18(69.2%)	8 (66.7% )	4.5	
Bronchiectasis	8(30.8%)	4 (33.35 )	3.8	
Severity of COVID19				
Mild low risk	283(75%)	53(54.6%)	1.75	0.025
Mild at risk	19(4.9%)	23(23.7%)	0.214	0.021
Moderate	31(8.1%)	11(11.3%)	2.86	0.001**
Severe	15(3.9%)	5(5.1%)	2.98	0.001**
Critical	30(7.8%)	3(3%)	3.24	0.001**
MV	24(6.2%)	2(2%)	4.12	0.001**
Died	16(4.1%)	1(1%)	3.4	0.01**

*HTN* hypertension, *DM* diabetes mellitus, *IHD* ischemic heart diseases, *CVA* cerebro-vascular stroke, *MV* mechanical ventilation

**:has significant level

## Discussion

The novel coronavirus disease (COVID-19, caused by the SARS-CoV-2 virus) has spread rapidly across the globe and has caused over 1,130,000 confirmed infections and over 62,000 deaths worldwide as of 5 April 2020 [6]. A number of risk factors for COVID-19 infection morbidity and mortality are known, including age, sex, and a number of chronic conditions and laboratory findings [1].

Biological factors that determine susceptibility to SARS-CoV-2 and severity of COVID-19 are yet to be fully understood. The ABO blood grouping may influence the susceptibility of COVID-19 and severity of the disease [7]. In this study, we found that ABO blood groups have different association risks for the infection with SARS-CoV-2 resulting in COVID-19. Specifically, blood group A was associated with an increased risk than blood group O. These findings are consistent with similar risk patterns of ABO blood groups for other coronavirus infections found in previous studies. For example, Cheng et al. reported that the SARS-CoV infection susceptibility in Hong Kong was differentiated by the ABO blood group systems [4]. The authors found that compared with non-O blood group hospital staff, blood group O hospital staff had a lower chance of getting infected. Many studies try to explain, and one study concluded that people with blood group O are able to recognize certain proteins as foreign and that may extend to proteins on virus surfaces so less likely to get a disease [8]. Another one found that anti-A antibodies inhibit binds of glycosylated SARS-COV S protein-expressing cells to angiotensin-converting enzyme 2 (ACE2) on cell surface, so these antibodies may block the interaction between the virus and its receptors thereby providing protection [9].

The activity of ACE2 in blood group B is much higher than in blood group O [10] which leads to catching the virus too much that may explain why critical patient and mortality was higher in blood group B than blood group O.

There was a link between blood group A and increase susceptibility of thromboembolism, diabetes mellitus, hypertension, recurrent urinary tract infections from *E. coli*, and *H. pylori* that can cause ulcers, stomach cancer, and heart diseases [11] so that another cause to explain why high mortality of COVID-19 in blood group A. Lastly there may be other mechanisms underlying why COVID-19 kills some people and spares others. HLAs represent just one in our immune system machinery, though, so their relative influence over COVID-19 infection remains unclear [12], noted that specific combinations of human leukocyte antigen (HLA) genes, which train immune cells to recognize germs, may be protective against SARS-CoV-2, while other combinations leave the body open to attack. Their analysis identified six HLA types with a high capacity to bind different SARS-CoV-2 protein sequences and three with a low capacity to do so. Specifically, a HLA type known as HLA-B*46:01 had the lowest predicted capacity to bind to bits of SARS-CoV-2 and associated with severe cases of infection. This speculation will need direct studies to prove it.

We believe that further longitudinal multi-center studies are highly needed to evaluate the clinical impact of those ABO groups in a single or multivariate analysis as a prognostic factor in COVID-19 patients.

## Conclusion

In this study, there was an association between the ABO blood group and COVID-19 susceptibility and severity. Specifically, people with blood group A have a higher susceptibility and severity whereas people with blood group O have a lower one.

People with blood group A:

1. Might need strengthened personal protection to reduce the chance of infection. And if infection was caught, might need to close observation and aggressive treatment.
2. It might be helpful to introduce ABO blood typing in the management of COVID-19 infection.

## Data Availability

All data of the patient is available on a computerized patient file in the Recording Department of King Fahd Hospital-Almadina Almonoura, KSA. www.KFHM-moh.gov.sa.

## Abbreviations

ACEP: American College of Emergency Physicians
ACE2: Angiotensin-converting enzyme 2
BMI: Body mass index
DM: Diabetes mellitus
HLA: Human leucocytic antigen
HTN: Hypertension
WHO: World Health Organization
